# Oxidative Stress in Alzheimer’s Disease: Can Dietary Interventions Provide Neuroprotection?

**DOI:** 10.3390/nu18152436

**Published:** 2026-07-25

**Authors:** Daria Kupczyk, Rafał Bilski, Igor Kozieł, Agata Słota, Mateusz Kurek, Emilia Stablewska, Szymon Baumgart, Artur Słomka, Renata Studzińska

**Affiliations:** 1 Department of Medical Biology and Biochemistry, Faculty of Medicine, Ludwik Rydygier Collegium Medicum in Bydgoszcz, Nicolaus Copernicus University in Toruń, 85-092 Bydgoszcz, Poland; dariak@cm.umk.pl; 2 Students Research Club of Medical Biology, Department of Medical Biology and Biochemistry, Faculty of Medicine, Ludwik Rydygier Collegium Medicum in Bydgoszcz, Nicolaus Copernicus University in Toruń, 87-100 Toruń, Poland; 3 Department of Organic Chemistry, Faculty of Pharmacy, Ludwik Rydygier Collegium Medicum in Bydgoszcz, Nicolaus Copernicus University in Toruń, 85-089 Bydgoszcz, Polandrstud@cm.umk.pl (R.S.); 4 Department of Hematology and Oncology, National Medical Institute of the Ministry of Interior and Administration, 02-507 Warsaw, Poland; artur.slomka@cm.umk.pl; 5 Department of Pathophysiology, Faculty of Pharmacy, Ludwik Rydygier Collegium Medicum in Bydgoszcz, Nicolaus Copernicus University in Toruń, 85-094 Bydgoszcz, Poland

**Keywords:** Alzheimer’s disease, oxidative stress, reactive oxygen species, antioxidants, diet, polyphenols, gut microbiota, gut–brain axis, neuroinflammation, neurodegeneration

## Abstract

Population aging is a growing problem. This process is driven not only by genetic factors but also by environmental factors, such as diet. Alzheimer’s disease (AD) is a progressive neurodegenerative disorder and the leading cause of dementia worldwide, characterized by cognitive decline, synaptic dysfunction, and neuronal loss. Despite extensive research, effective disease-modifying therapies remain limited. Increasing evidence indicates that oxidative stress plays a central role in AD pathogenesis, acting as a key link between β-amyloid accumulation, tau hyperphosphorylation, mitochondrial dysfunction, and neuroinflammation. Accordingly, dietary strategies have been proposed to mitigate these pathological processes and may represent an important component of Alzheimer’s disease prevention. Moreover, emerging evidence on the gut–brain axis highlights the critical role of gut microbiota in regulating neuroinflammation and oxidative stress. Dysbiosis has been associated with increased permeability of the intestinal barrier, systemic inflammation, and accelerated neurodegeneration. Dietary patterns such as the Mediterranean, DASH, and MIND diets may exert beneficial effects by simultaneously influencing antioxidant status and microbial composition. This review aims to provide a comprehensive overview of the role of oxidative stress in Alzheimer’s disease and evaluate the potential of dietary interventions in modulating mechanisms involved in Alzheimer’s disease pathogenesis and supporting cognitive health. Particular attention is given to the neuroprotective effects of dietary antioxidants, including vitamins, polyphenols, and polyunsaturated fatty acids, which act through the reduction in reactive oxygen species, modulation of inflammatory pathways, and support of neuronal survival. Although current findings are promising, inconsistencies in clinical data indicate the need for further well-designed studies. Future research should focus on personalized nutritional strategies integrating dietary, genetic, and microbiome-related factors. Targeting oxidative stress through diet and microbiota modulation represents a promising complementary strategy for Alzheimer’s disease prevention and supportive management, although further clinical studies are required to establish disease-modifying effects.

## 1. Introduction

The epidemiology of neurodegenerative diseases is linked to the aging of the population. According to data from the World Health Organization (WHO), the number of people living with dementia worldwide exceeds 55 million, and projections indicate that this number will increase by 2050. The prevalence of Alzheimer’s disease (AD) in people aged 60 is 1%, and after the age of 65, it increases to 5% [1,2]. It is characterized by a progressive decline in cognitive functions, including memory, language, and executive abilities, ultimately leading to a loss of independence and severe impairment in daily functioning. The global burden of AD is steadily increasing, both in terms of prevalence and socioeconomic costs, emphasizing the urgent need for effective preventive and therapeutic strategies [3,4]. Despite decades of intensive research, Alzheimer’s disease remains incurable. Traditional pharmacological treatments, including acetylcholinesterase inhibitors and memantine, primarily provide symptomatic benefit. More recently, anti-amyloid monoclonal antibodies have become available for selected patients with early symptomatic Alzheimer’s disease and confirmed amyloid pathology. Although these therapies can modify amyloid-related biomarkers and may modestly slow cognitive and functional decline in appropriately selected patients, their clinical applicability is limited by eligibility requirements, monitoring needs, adverse effects, costs, and differences in regulatory availability. Therefore, increasing attention has been directed toward modifiable risk factors, including lifestyle and dietary habits, which may influence disease risk and pathogenic mechanisms [1,5,6]. The pathogenesis of AD is complex and multifactorial, involving the accumulation of β-amyloid (Aβ) plaques, neurofibrillary tangles composed of hyperphosphorylated tau protein, neuroinflammation, and synaptic dysfunction. Among the various mechanisms proposed, oxidative stress has emerged as a central and unifying factor linking multiple pathological pathways [7,8,9]. These changes lead to the loss of interneuronal connections and neuronal death. There is no single theory that explains the aging process and the development of age-related diseases. Current evidence indicates that oxidative stress not only contributes to neuronal damage but also promotes amyloid aggregation, tau hyperphosphorylation, and neuroinflammatory responses. In recent years, growing attention has been paid to the role of diet and nutritional interventions in modulating oxidative stress and neurodegeneration. An additional emerging area of interest is the role of the gut microbiota and its interaction with the central nervous system through the gut–brain axis.

This narrative review critically examines oxidative stress as an integrative mechanism linking mitochondrial dysfunction, neuroinflammation, amyloid and tau pathology in Alzheimer’s disease. It further evaluates the extent to which dietary patterns, dietary antioxidants, and microbiota-related mechanisms may modify these processes, with particular emphasis on distinguishing preclinical findings from evidence obtained in human observational and interventional studies. Unlike previous reviews focusing separately on oxidative stress, dietary antioxidants, or the gut microbiota, the present review integrates these domains within a single diet–microbiota–redox framework and critically evaluates their translational relevance.

## 2. Literature Search Strategy

This narrative review was based on literature searches conducted in PubMed/MEDLINE, Scopus, and Web of Science up to 10.06.2026. Search terms included combinations of “Alzheimer’s disease”, “oxidative stress”, “reactive oxygen species”, “diet”, “antioxidants”, “Mediterranean diet”, “MIND diet”, “gut microbiota”, “probiotics”, and “gut–brain axis”. When multiple publications addressed the same topic, preference was given to recent systematic reviews, meta-analyses, and randomized controlled trials, whereas preclinical studies were primarily used to explain biological mechanisms. Preclinical studies were included when human evidence was unavailable or when they provided mechanistic context. Evidence was synthesized narratively and categorized as clinical, observational, preclinical, or hypothetical. Publications written in English and directly addressing oxidative stress, dietary interventions, gut microbiota, or Alzheimer’s disease were considered eligible. Studies were screened for relevance based on titles, abstracts, and full-text evaluation, with priority given to high-quality clinical evidence and recent mechanistic investigations. Conference abstracts, editorials, letters, and studies not directly relevant to the scope of the review were excluded.

## 3. Pathophysiology of Alzheimer’s Disease

Genetic predisposition plays a significant role in the development of Alzheimer’s disease, particularly in early-onset forms [10]. One of the key genes involved is the amyloid precursor protein (APP) gene, which encodes a transmembrane protein that undergoes enzymatic processing. Under pathological conditions, APP is cleaved by β-secretase (BACE1) and γ-secretase, leading to the formation of β-amyloid peptides, particularly Aβ42, which exhibit a high propensity for aggregation and toxicity [11,12]. This may also lead to a reduction in the amount of the messenger substance acetylcholine [13]. These Aβ peptides accumulate in the extracellular space and may form senile plaques; however, current interpretations of Alzheimer’s disease no longer regard plaque deposition as a simple, linear cause of neurodegeneration. Contemporary revisions of the amyloid cascade hypothesis emphasize that soluble Aβ species, particularly oligomers and protofibrils, may be more biologically relevant than mature plaques, while plaque burden itself correlates only imperfectly with cognitive decline. Although Aβ remains an important pathological hallmark and therapeutic target, recent evidence suggests that it is unlikely to act as the sole primary driver of Alzheimer’s disease [14]. Instead, Aβ pathology is increasingly viewed as one component of a broader, multifactorial disease network involving tau pathology, synaptic dysfunction, neuroinflammation, oxidative stress, vascular and metabolic abnormalities, impaired proteostasis, and altered glial–neuronal interactions. In this context, anti-amyloid therapies may support a contributory role for Aβ in disease progression, particularly in early Alzheimer’s disease, but their modest clinical effects and safety concerns indicate that amyloid removal alone does not fully address the underlying etiological complexity of the disorder. Alternative or complementary models, including presenilin loss-of-function mechanisms and impaired astrocytic glutamate clearance via Excitatory amino acid transporter 2 (EAAT2), further suggest that synaptic failure and excitotoxicity may participate in neurodegeneration independently of, or downstream from, amyloid accumulation [15,16]. Another critical genetic factor is the apolipoprotein E (APOE) gene, which is considered the strongest genetic risk factor for late-onset AD. The APOE ε4 allele is associated with increased Aβ aggregation and impaired clearance from the brain, thereby accelerating plaque formation and disease progression [17]. In a gene dose-dependent manner, carrying one ε4 allele increases the risk of AD by approximately 3–7-fold, whereas homozygous carriers may exhibit up to a 12–15-fold higher risk compared with non-carriers [17,18]. APOE ε4 is also associated with an earlier age of disease onset, reducing it by approximately 7–9 years per allele copy [18]. Beyond its effects on amyloid metabolism, APOE4 has been implicated in tau hyperphosphorylation, neuroinflammation, lipid dysregulation, mitochondrial dysfunction, and blood–brain barrier impairment, suggesting that its contribution to AD pathogenesis extends well beyond amyloid plaque formation [17,18]. In contrast, the APOE ε2 allele appears to exert a protective effect against AD, while APOE ε3 is considered the neutral and most common isoform in the general population [17,19].

In addition to amyloid pathology, tau protein abnormalities play a central role in AD pathogenesis. Tau is a microtubule-associated protein responsible for stabilizing cytoskeletal structures and facilitating axonal transport. In Alzheimer’s disease, tau undergoes hyperphosphorylation, which reduces its affinity for microtubules and promotes its aggregation into paired helical filaments and neurofibrillary tangles [20]. The formation of neurofibrillary tangles disrupts intracellular transport mechanisms, leading to impaired synaptic function and neuronal degeneration. Unlike amyloid plaques, which accumulate extracellularly, tau pathology spreads intracellularly in a prion-like manner, correlating strongly with disease severity and cognitive decline [21,22,23].

Neuroinflammation is another key component of AD pathophysiology. The accumulation of Aβ and tau pathology activates microglia and astrocytes, leading to the release of pro-inflammatory cytokines such as interleukin-1β (IL-1β) and tumor necrosis factor-α (TNF-α). Chronic activation of these immune responses contributes to neuronal damage and accelerates disease progression. In parallel, synaptic dysfunction represents one of the earliest events in AD. Aβ oligomers interfere with synaptic transmission and plasticity, particularly affecting cholinergic signaling pathways, which are essential for memory and learning. This disruption contributes to the early cognitive deficits observed in patients [24]. Unfortunately, the existing inflammatory state promotes excessive production of reactive oxygen (ROS) and nitrogen species (RNS), thereby intensifying lipid peroxidation processes in neuronal cell membranes [25]. This oxidative damage compromises membrane integrity, alters ion channel and receptor function, and disrupts mitochondrial activity, further impairing neuronal homeostasis. In particular, neurons are highly vulnerable to oxidative stress due to their elevated metabolic demand and lipid-rich membranes. Therefore, oxidative membrane damage exacerbates synaptic dysfunction and contributes to progressive neurodegeneration in Alzheimer’s disease [26]. Although multiple hypotheses have been proposed to explain AD pathogenesis, increasing evidence suggests that mitochondrial dysfunction and oxidative stress serve as central integrative mechanisms linking these processes. Aβ accumulation, tau pathology, and neuroinflammation collectively contribute to increased production of ROS, leading to cellular damage and neuronal death [27]. Mitochondrial impairment results in decreased adenosine triphosphate (ATP) production, disruption of calcium homeostasis, and increased oxidative damage, further exacerbating neurodegeneration. These processes create a vicious cycle in which oxidative stress amplifies amyloid and tau pathology, ultimately accelerating disease progression [28].

## 4. Oxidative Stress in Alzheimer’s Disease

Increasing with age, oxidative stress is widely recognized as one of the central mechanisms contributing to the pathogenesis and progression of Alzheimer’s disease [29]. Oxidative stress is defined as a condition where pro-oxidative activities outweigh cellular antioxidant defense because redox signaling and adaptability are disrupted [30]. This imbalance leads to cumulative damage of cellular components, including lipids, proteins, and nucleic acids, ultimately contributing to neuronal dysfunction and death. Reactive oxygen species are generated as natural by-products of cellular metabolism, primarily within mitochondria during oxidative phosphorylation. Under physiological conditions, ROS play essential roles in intracellular signaling, regulation of gene expression, and modulation of apoptosis [31,32]. However, excessive ROS production or impaired antioxidant defenses result in oxidative stress, which disrupts cellular homeostasis. Major sources of ROS in the central nervous system include mitochondrial electron transport chain leakage, activation of nicotinamide adenine dinucleotide phosphate (NADPH) oxidases, and enzymatic reactions involving oxidases and peroxidases. External factors, such as environmental toxins, radiation, and metabolic disturbances, may further enhance ROS production [33]. Mitochondrial dysfunction is a hallmark of Alzheimer’s disease and a major contributor to oxidative stress [34]. In AD, β-amyloid peptides accumulate within mitochondria, where they interfere with key components of the electron transport chain, particularly cytochrome c oxidase (complex IV). This disruption leads to impaired electron transfer and increased leakage of electrons, which react with molecular oxygen to form superoxide radicals [35]. The accumulation of ROS within mitochondria results in lipid peroxidation, protein oxidation, and mitochondrial DNA damage, further impairing mitochondrial function [36,37,38]. Additionally, excessive ROS production promotes mitochondrial fragmentation through dysregulation of fission–fusion dynamics, leading to structurally and functionally compromised organelles [39]. A critical event associated with oxidative stress is the opening of the mitochondrial permeability transition pore (mPTP), which is triggered by oxidation of thiol groups in regulatory proteins such as cyclophilin D. Opening of the mPTP results in loss of mitochondrial membrane potential, influx of ions, mitochondrial swelling, and release of pro-apoptotic factors, including cytochrome c and apoptosis-inducing factor (AIF), into the cytoplasm [40]. This process activates caspase-dependent and caspase-independent apoptotic pathways, ultimately leading to neuronal cell death [41,42].

The brain is particularly vulnerable to oxidative damage because of its high oxygen consumption, abundant polyunsaturated lipids, relatively limited antioxidant capacity, and high metabolic activity. Although the blood–brain barrier normally restricts the entry of many potentially harmful circulating substances, its integrity and transport functions may be impaired during aging, systemic inflammation, and Alzheimer’s disease, potentially increasing neuronal exposure to inflammatory and oxidative damage [43]. Excessive ROS levels contribute to widespread damage of cellular macromolecules. Lipid peroxidation is particularly prominent in the brain due to the high content of polyunsaturated fatty acids in neuronal membranes [44]. This process leads to the formation of toxic by-products, such as malondialdehyde (MDA) and 4-hydroxy-2-nonenal (4-HNE), which further disrupt membrane integrity and protein function [45]. Proteins are also highly susceptible to oxidative modification, resulting in structural alterations, loss of enzymatic activity, and increased susceptibility to aggregation [46]. Oxidative damage to nucleic acids, including the formation of 8-oxo-deoxyguanosine (8-oxo-dG), contributes to genomic instability and impaired cellular repair mechanisms [47,48]. Clinical studies have demonstrated elevated levels of oxidative stress biomarkers in the brains and cerebrospinal fluid of AD patients, supporting the role of oxidative damage in disease progression (Table 1).

**Table 1:** Oxidative, nitrosative, and carbonyl stress biomarkers associated with Alzheimer’s disease and the type of evidence supporting their role in neurodegeneration.

Oxidative Stress Marker	Changes in AD	Biological Material	Type of Study	Significance in AD	References
MDA	Increased	Cerebrospinal fluid (CSF), serum, plasma, brain tissue	Clinical studies + in vivo studies	Major marker of lipid peroxidation indicating oxidative membrane damage and early neurodegenerative changes.	[49,50,51,52]
4-HNE	Increased	Brain tissue, AD models	Postmortem studies + in vivo studies	Toxic lipid peroxidation product involved in synaptic dysfunction, protein modification, and neuronal apoptosis.	[53,54]
F2-isoprostanes	Increased	CSF, blood, brain tissue	Clinical studies	Reliable indicator of oxidative lipid damage and neuronal membrane degeneration.	[55,56]
8-oxo-dG	Increased	CSF, blood, brain tissue	Clinical studies	Marker of oxidative DNA damage associated with neuronal genomic instability and disease progression.	[57,58,59]
Advanced oxidation protein products (AOPP)	Increased	Plasma	Clinical case–control studies	Reflect oxidative protein modification and systemic oxidative stress linked to neurodegeneration.	[60,61]
Protein carbonyls	Increased	Plasma, CSF, brain tissue	Clinical studies	Indicators of irreversible protein oxidation contributing to impaired neuronal function and tau pathology.	[62,63]
3-Nitrotyrosine	Increased	CSF, brain tissue	Clinical/postmortem studies + in vivo studies	Marker of nitrosative stress and peroxynitrite-mediated protein damage in AD brains.	[64,65,66]
Advanced glycation end products (AGEs)	Increased	Plasma, brain tissue	Clinical studies	Promote oxidative stress, inflammation, and Aβ aggregation through Receptor for Advanced Glycation Endproducts (RAGE) signaling pathways.	[67,68,69]

**Figure 1:**
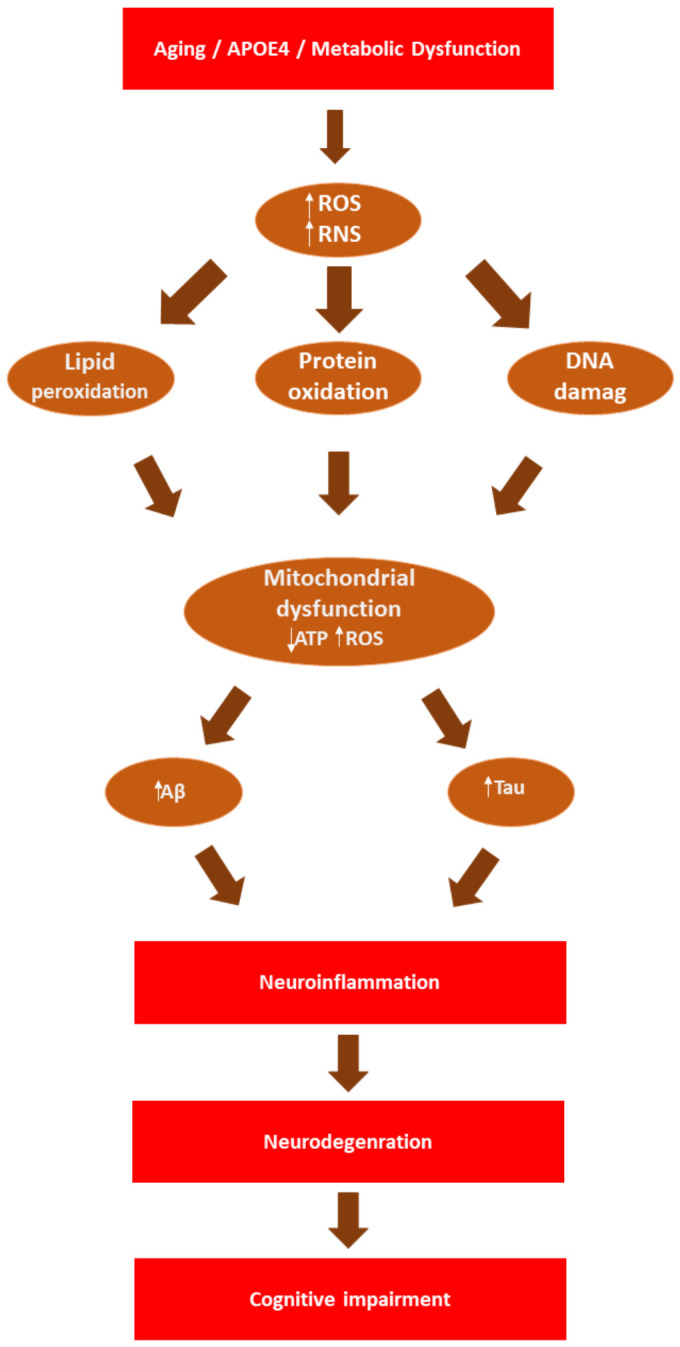
Oxidative Stress–Driven Mechanisms of Neurodegeneration in Aging.

Oxidative stress not only results from pathological processes in AD but also actively contributes to their progression. It has been shown to enhance the amyloidogenic processing of APP by increasing β-secretase activity while reducing α-secretase activity, thereby promoting Aβ production and aggregation [70]. Furthermore, oxidative stress stimulates kinases such as glycogen synthase kinase-3β (GSK-3β), which promotes tau hyperphosphorylation and the formation of neurofibrillary tangles. These pathological changes disrupt microtubule stability and impair axonal transport, exacerbating neuronal dysfunction [71]. The role of oxidative stress in neurodegeneration is summarized in Figure 1.

Additionally, oxidative stress activates microglia and induces the release of pro-inflammatory cytokines, linking oxidative damage with neuroinflammation. Metal ions, such as iron and copper, may further amplify ROS production through Fenton reactions, creating a feedback loop that accelerates neurodegeneration [72]. To counteract oxidative stress, cells possess complex antioxidant defense mechanisms, which include both enzymatic and non-enzymatic components. Key enzymatic antioxidants include superoxide dismutase (SOD), catalase (CAT), and glutathione peroxidase (GSH-Px), which neutralize ROS and prevent oxidative damage [8]. Non-enzymatic antioxidants, such as vitamins C and E, carotenoids, and polyphenols, play a complementary role by scavenging free radicals and maintaining redox balance. However, in Alzheimer’s disease, these defense systems are often overwhelmed or impaired, leading to sustained oxidative stress and progressive neuronal damage [8]. Ageing is associated with a progressive decline in endogenous antioxidant defense mechanisms.

## 5. Dietary Antioxidants and Neuroprotection in Alzheimer’s Disease

### 5.1. Individual Dietary Components

Growing evidence suggests that dietary factors may influence the risk of Alzheimer’s disease and several biological mechanisms involved in its pathogenesis. In particular, antioxidants derived from diet have attracted considerable attention due to their ability to counteract oxidative stress, reduce neuroinflammation, and support neuronal survival. These compounds may influence multiple pathological pathways involved in AD, including amyloid aggregation, tau hyperphosphorylation, and mitochondrial dysfunction [73]. Dietary antioxidants exert neuroprotective effects through several complementary mechanisms. Their primary function involves the neutralization of ROS, thereby reducing oxidative damage to lipids, proteins, and nucleic acids. In addition, antioxidants modulate intracellular signaling pathways associated with inflammation, apoptosis, and cellular survival [74]. Importantly, antioxidants have been shown to regulate key molecular pathways such as nuclear factor erythroid 2-related factor 2 (Nrf2), nuclear factor kappa B (NF-κB), and mitogen-activated protein kinase (MAPK), which are involved in maintaining redox balance and controlling inflammatory responses. Through these pathways, antioxidants can attenuate neuroinflammation and promote neuronal resilience [74,75,76]. Furthermore, certain antioxidant compounds can chelate metal ions such as iron and copper, thereby reducing ROS generation through Fenton reactions. This is particularly relevant in AD, where metal dyshomeostasis contributes to oxidative stress and amyloid toxicity [77]. Another important antioxidant is glutathione, which plays a key role in the regeneration of vitamins C and E [76]. Maintaining adequate intracellular glutathione levels may support antioxidant defense and neuronal homeostasis; however, its clinical effect on AD progression remains uncertain. Vitamins C and E are among the most extensively studied dietary antioxidants in the context of Alzheimer’s disease [78]. Vitamin E, a lipid-soluble antioxidant, plays a crucial role in protecting neuronal membranes from lipid peroxidation. It acts by interrupting free radical chain reactions within lipid bilayers, thereby preserving membrane integrity and function. Vitamin C, a water-soluble antioxidant, contributes to the regeneration of oxidized vitamin E and directly scavenges free radicals [79,80]. Observational clinical studies suggest that higher dietary intake of vitamin C is associated with a reduced risk of AD, particularly at intake levels above 75 mg per day. However, findings regarding vitamin E and β-carotene remain inconsistent, indicating that the effectiveness of supplementation may depend on dosage, bioavailability, and interactions with other nutrients [81]. Polyphenols represent a diverse group of plant-derived compounds with strong antioxidant and anti-inflammatory properties. They are widely found in fruits, vegetables, tea, coffee, and cocoa. Common polyphenols include flavonoids, resveratrol, curcumin, and catechins [82]. These compounds exert neuroprotective effects through multiple mechanisms, including inhibition of Aβ aggregation, reduction in tau hyperphosphorylation, and modulation of synaptic plasticity. Preclinical studies have shown that polyphenols activate cytoprotective pathways, such as Nrf2 signaling, and enhance the expression of brain-derived neurotrophic factor (BDNF), which supports neuronal survival and plasticity [83,84,85]. In addition, polyphenols exhibit anti-inflammatory properties by suppressing pro-inflammatory cytokine production and inhibiting microglial activation [86]. These findings suggest that polyphenols may represent promising candidates for prevention and supportive management of AD. However robust clinical confirmation remains limited. Polyunsaturated fatty acids (PUFAs), particularly omega-3 fatty acids, play an essential role in maintaining neuronal membrane structure and function. They are involved in synaptic plasticity, neurotransmission, and anti-inflammatory processes [87]. The preclinical trials reported that omega-3 fatty acids have been shown to reduce neuroinflammation, modulate oxidative stress, and support cognitive function. However, the results of clinical trials are contradictory. Their incorporation into neuronal membranes enhances membrane fluidity and may improve signal transmission [87]. Additionally, PUFAs may influence amyloid metabolism and reduce Aβ accumulation, although clinical evidence remains inconclusive [88,89,90]. Vitamins play a crucial role in the growth, development, and differentiation of nervous system cells [91,92,93,94].

### 5.2. Dietary Patterns and Emerging Nutritional Strategies

Ageing is associated with progressive deterioration of gastrointestinal function. Intestinal motility decreases and chewing becomes more difficult. Protein and nutrient deficiencies develop, calorie deficits increase, and vitamin and nutrient absorption become more difficult. Furthermore, age-related metabolic diseases develop [95]. Therefore, a diet should be well-balanced, varied, easily digestible, and free from large amounts of simple sugars and processed foods, but rich in fiber [96]. Recommended dietary patterns include the Mediterranean diet, which includes leafy vegetables, fresh fruit, and olive oil [97]. This diet limits the consumption of animal fats. Another recommended dietary pattern is the DASH (Dietary Approaches to Stop Hypertension) diet, which limits salt and red meat consumption. Along with the Mediterranean diet, it is currently recommended for its greatest health benefits [98]. The fasting-mimicking diet (FMD) is one of the emerging research directions for nutritional neuroprotective strategies. Unlike traditional caloric restriction, FMD is based on periodic consumption of a reduced-energy diet with a modified macronutrient composition, which allows for the activation of multiple metabolic pathways associated with the response to nutrient deprivation. Preclinical studies indicate that this diet can reduce oxidative stress, reduce neuroinflammation, stimulate autophagy, and improve mitochondrial function and energy homeostasis in neurons. Furthermore, it has been suggested that this diet may influence the composition and metabolic activity of the gut microbiota, potentially enhancing its beneficial effects on the gut–brain axis. However, most available data come from animal models, while clinical evidence in Alzheimer’s disease is still limited and consists primarily of early studies assessing the safety and feasibility of this type of intervention. Therefore, the effectiveness of the fasting-mimicking diet in preventing or treating Alzheimer’s disease requires confirmation in well-designed, randomized clinical trials [99].

However, the recommended dietary model for Alzheimer’s disease prevention is the MIND diet, which includes high levels of docosahexaenoic acid (DHA). DHA has been associated with possible reduced amyloid pathology in experimental studies. A high intake of vitamins in the diet helps combat oxidative stress and reduce inflammation. The MIND diet includes foods that have a beneficial effect on brain function. These include berries, a source of polyphenols, green leafy vegetables, due to their high vitamin C content, and antioxidant-rich nuts, whole grains, fish, olive oil, and legumes. Therefore, it is a diet rich in antioxidant compounds, dietary fiber, and unsaturated fatty acids [100,101,102]. Rather than focusing solely on individual nutrients, recent research emphasizes the importance of overall dietary patterns. Diets rich in antioxidant and anti-inflammatory components have been associated with a reduced risk of cognitive decline and Alzheimer’s disease. The Mediterranean diet, characterized by high intake of fruits, vegetables, legumes, whole grains, and olive oil, has demonstrated the most consistent protective effects. Similarly, the DASH and MIND diets, which emphasize plant-based foods and limit saturated fats, have shown promising results in reducing AD risk and slowing cognitive decline in observational studies, although evidence from clinical intervention trials remains limited. Among currently studied dietary patterns, the MIND diet appears to have the strongest epidemiological support in relation to AD prevention. Its practical advantages include ease of implementation, cultural adaptability, and suitability for long-term use in older adults. The summary of beneficial effects and the limitations of different diets in AD is presented in Table 2.

**Table 2:** Benefits and limitations of different diets in AD.

Diet	Cognitive Benefits	Limitations/Negative Effects	Mechanisms/Key Observations	References
Mediterranean Diet	Associated with a lower risk of Alzheimer’s disease; decreased cognitive decline; improved cognitive performance	No major adverse cognitive effects reported	Anti-inflammatory effects; improved vascular health; may reduce amyloid-beta burden	[103,104,105,106]
DASH Diet	Potential improvement in cognitive function; reduced risk factors associated with cognitive decline	No consistent findings; no significant associations with cognitive outcomes in some studies; weaker evidence compared to Mediterranean diet	Blood pressure regulation; cardiovascular protection	[107,108,109]
MIND Diet	Decreased cognitive decline; promising neuroprotective potential; possibly more effective than Mediterranean or DASH alone	Limited long-term clinical evidence	Targeted neuroprotective dietary components (berries, leafy greens)	[100,101,110,111,112,113,114]
Ketogenic Diet	Potential improvement in cognitive function in AD patients	Limited and inconsistent evidence; unclear long-term effects	Alternative brain energy metabolism via ketone bodies	[115,116,117,118]
Plant-based Diets	Possibly decreased cognitive decline; associated with improved cognitive resilience	Variability depending on dietary composition and adherence	Reduced inflammation; improved metabolic and cardiovascular health	[119,120,121]
Western Diet	No cognitive benefits reported	Increased cognitive decline; increased risk of Alzheimer’s disease; impaired cognitive function	Increased intake of saturated fats and processed foods; associated with increased inflammation and insulin resistance	[122,123,124,125]
High saturated fat/processed food diets	No cognitive benefits reported	Increased risk of cognitive impairment and dementia	Increased oxidative stress; increased neuroinflammation	[126,127,128]
Diets rich in neuroprotective nutrients (PUFA, vitamins, antioxidants)	May support cognitive function; slowed disease progression	Evidence is still limited and sometimes inconsistent	Reduced oxidative stress; improved neuronal and synaptic function	[87,129,130,131]

These dietary patterns provide a synergistic combination of antioxidants, vitamins, and bioactive compounds that collectively support brain health. Their effects are likely mediated through the reduction in oxidative stress, improvement of vascular function, and modulation of inflammatory pathways.

## 6. Gut Microbiota and the Gut–Brain Axis in Alzheimer’s Disease

**Figure 2:**
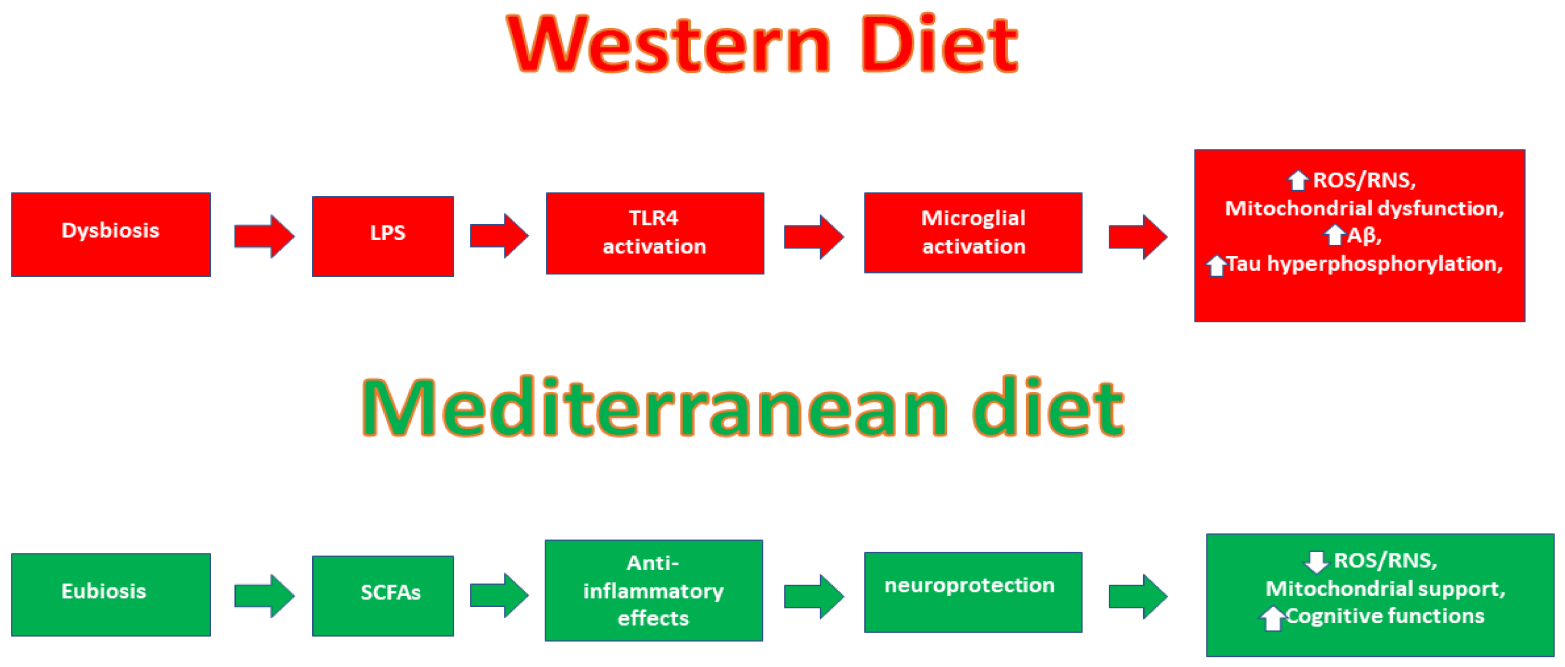
Proposed mechanisms linking Western and Mediterranean dietary patterns to Alzheimer’s disease through modulation of the gut microbiota–brain axis.

In recent years, the gut microbiota has emerged as a critical factor influencing the development and progression of neurodegenerative diseases, including AD. The complex bidirectional communication system between the gastrointestinal tract and the central nervous system, known as the gut–brain axis, integrates neural, immune, endocrine, and metabolic pathways [132,133]. Disruptions within this system have been increasingly implicated in the pathogenesis of AD. The gut–brain axis is a dynamic and bidirectional network that enables communication between the gut microbiota and the central nervous system. This interaction occurs through multiple interconnected pathways, including neural signaling mediated by the vagus nerve and the enteric nervous system, immune-related mechanisms involving cytokine production, endocrine communication through hormones and neurotransmitters, and metabolic processes associated with microbial metabolites. The gut microbiota is a crucial link between diet and oxidative stress in Alzheimer’s disease. Beneficial bacterial metabolites, especially short-chain fatty acids (SCFAs), support endogenous antioxidant mechanisms by activating the Nrf2 pathway and simultaneously inhibiting the NF-κB pathway, which leads to reduced production of ROS and attenuated inflammatory responses. Dysbiosis, in turn, promotes intestinal barrier damage, increased inflammation, and increased oxidative stress, contributing to the progression of neurodegeneration. This suggests that the neuroprotective effects of diets such as the Mediterranean diet or MIND may result not only from the direct antioxidant effects of their components but also from beneficial modulation of the composition and metabolic activity of the gut microbiota. These observations support a proposed mechanistic axis linking diet, the gut microbiota, Nrf2/NF-κB signaling, oxidative stress, and neuroprotective processes. Gut microbiota contributes significantly to the production of neurotransmitters such as serotonin and dopamine, which play essential roles in cognitive function and emotional regulation [134]. These interactions underscore the importance of the microbiome in maintaining neurological homeostasis. Dysbiosis, defined as an imbalance in the composition and function of gut microbiota, has been associated with increased risk of Alzheimer’s disease. Alterations in microbial diversity can lead to disruption of the intestinal barrier, allowing microbial products such as lipopolysaccharides (LPS) to enter systemic circulation and trigger inflammatory responses [135]. These processes contribute to chronic low-grade inflammation and increased oxidative stress, both of which are key factors in AD progression. Dysbiosis has also been linked to enhanced activation of microglia, the resident immune cells of the brain, which further amplifies neuroinflammatory processes. One of the primary mechanisms through which gut microbiota influence brain function is the production of metabolites, particularly SCFAs, such as acetate, propionate, and butyrate. These compounds play a crucial role in maintaining intestinal barrier integrity, modulating immune responses, and influencing brain function [135,136]. SCFAs have been shown to exert anti-inflammatory effects, regulate microglial activity, and support neuronal survival. Conversely, reduced production of beneficial metabolites due to dysbiosis may contribute to increased neuroinflammation and oxidative stress, thereby accelerating neurodegeneration [137]. Furthermore, gut microbiota can influence the metabolism of dietary compounds, including polyphenols, enhancing their bioavailability and biological activity. This interaction underscores the close relationship between diet, microbiota, and brain health. The relationship between gut microbiota and Alzheimer’s disease is closely linked to oxidative stress and neuroinflammation. Dysbiosis has been associated with impaired intestinal barrier function, increased inflammation, and oxidative stress, which may contribute to neurodegenerative processes. This results in a self-perpetuating cycle in which oxidative stress and inflammation reinforce each other, leading to progressive neuronal damage [136,137]. Moreover, alterations in microbiota composition have been associated with impaired regulation of key signaling pathways, including NF-κB and Nrf2, which are involved in inflammatory responses and antioxidant defense mechanisms [138]. The comparison of influence of Western and Mediterranean diet on AD is shown in Figure 2.

Given the significant role of gut microbiota in the pathogenesis of AD, microbiota-targeted interventions have gained increasing attention as potential therapeutic strategies. These approaches include dietary modifications aimed at promoting beneficial microbial composition, supplementation with probiotics and prebiotics, and the use of bioactive compounds with microbiota-modulating properties [139]. Dietary patterns rich in fiber, polyphenols, and unsaturated fatty acids have been shown to support microbial diversity and promote the production of beneficial metabolites. Such interventions may reduce oxidative stress, attenuate neuroinflammation, and improve cognitive outcomes. Emerging evidence suggests that modulation of the gut microbiota may influence mechanisms associated with AD pathogenesis and could contribute to preventive or supportive strategies but also serve as a preventive strategy by targeting early pathological processes before the onset of clinical symptoms [140,141]. The summary of bacterial taxa with beneficial effects on cognition in AD is shown in Table 3.

**Table 3:** Selected bacterial taxa with reported beneficial effects on cognition and Alzheimer’s disease pathology.

Bacterial Taxa/Strain	Model	Observed Effect on Cognition/AD	Proposed Mechanism	References
*Bifidobacterium breve A1*	Animal model, Human studies	Preliminary improvement (including memory and Mini-Mental State Examination (MMSE) scores) reported in preclinical and early clinical studies	Decreased neuroinflammation, modulation of immune-related gene expression, potential reduction in Aβ pathology	[142,143,144]
*Bifidobacterium bifidum + Bifidobacterium longum*	Animal model, Human studies	Preliminary Improvement in cognitive function and mental state in early clinical trials	Modulation of gut microbiota, possible effects on BDNF and inflammatory pathways	[145,146,147,148]
*Lactobacillus plantarum*	Animal model	Attenuated cognitive deficits reported in preclinical studies	Decreased amyloid-beta and tau pathology, decreased oxidative stress	[149,150,151,152]
*Clostridium butyricum*	Animal model	Attenuated cognitive decline reported in preclinical studies	Increased butyrate production, decreased microglial activation, decreased pro-inflammatory cytokines, decreased Aβ accumulation	[153,154,155]
*Escherichia coli Nissle 1917*	Animal model, Human studies	Improved MMSE scores after intervention, reported in preclinical and clinical studies	Decreased oxidative stress, decreased inflammation, improved gut barrier integrity	[156,157]
*Akkermansia muciniphila*	Animal model	improved cognition in preclinical models	Modulation of gut barrier, SCFA production, inflammation regulation	[158,159,160,161]

## 7. Discussion

**Figure 3:**
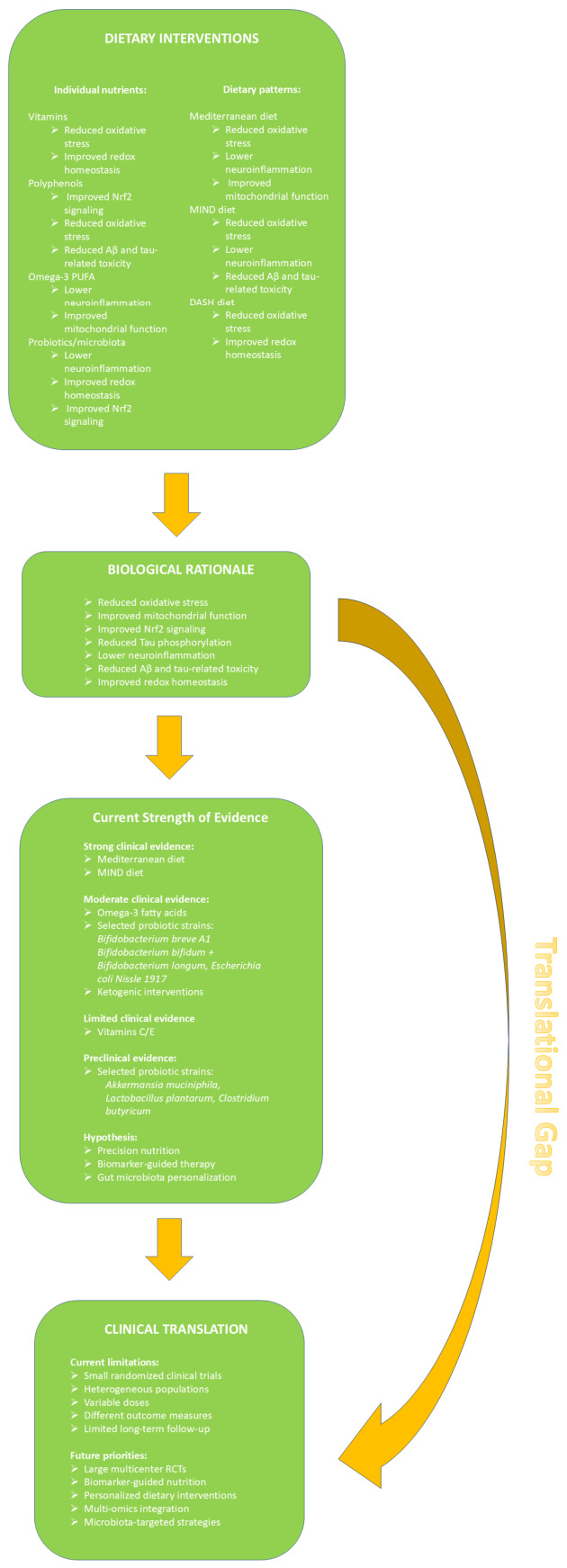
Summary of the current evidence supporting dietary interventions targeting oxidative stress in Alzheimer’s disease.

Oxidative stress emerges from the reviewed evidence as an integrative, bidirectional mechanism linking mitochondrial dysfunction, amyloid and tau pathology, and neuroinflammation. However, its central role does not imply that antioxidant interventions will necessarily produce clinically meaningful benefits, as demonstrated by the inconsistent results of human supplementation trials. As summarized in Figure 3, the current body of evidence demonstrates a substantial gap between promising mechanistic findings and their translation into clinical practice.

However, despite promising preclinical data, clinical evidence remains inconsistent. While some studies suggest that higher dietary intake of antioxidants, particularly vitamin C, may reduce the risk of AD, others have failed to demonstrate significant benefits of supplementation. These discrepancies may be explained by differences in study design, dosage, bioavailability, and interactions between nutrients. Importantly, recent research has shifted focus from individual nutrients toward overall dietary patterns. Diets such as the Mediterranean, DASH, and MIND diets provide a synergistic combination of bioactive compounds that may collectively exert neuroprotective effects. This holistic approach reflects the complexity of AD pathogenesis and suggests that single-target interventions may be insufficient to significantly alter disease progression. The emerging role of the gut microbiota further expands this perspective by introducing the gut–brain axis as a critical mediator between diet and brain function. Dysbiosis has been linked to increased oxidative stress, systemic inflammation, and disruption of the blood–brain barrier, all of which contribute to neurodegeneration. Microbial metabolites, particularly short-chain fatty acids, play a key role in modulating immune responses and maintaining neuronal homeostasis. From a public health perspective, dietary interventions represent an attractive preventive strategy because they are relatively safe, inexpensive, and applicable at the population level. Unlike pharmacological approaches, dietary modifications can target multiple pathogenic pathways simultaneously, including oxidative stress, inflammation, vascular dysfunction, and microbiota-related mechanisms. The interaction between diet, microbiota, and oxidative stress represents a promising area for future research. Dietary components not only exert direct antioxidant effects but also indirectly influence brain health by shaping the composition and metabolic activity of the gut microbiota. This dual mechanism may enhance the effectiveness of dietary interventions and provide a more comprehensive approach to disease prevention and management. This review focuses on presenting oxidative stress as a mechanism linking the classic elements of Alzheimer’s disease pathogenesis with the influence of environmental factors, particularly diet and the gut–brain axis. Accumulating evidence indicates that oxidative stress is not merely a secondary consequence of β-amyloid deposition or tau protein hyperphosphorylation, but rather represents a key mechanism that exacerbates mitochondrial dysfunction, chronic inflammation, gut microbiota dysfunction, and progressive neuronal damage. This approach to pathogenesis suggests that nutritional interventions may exert neuroprotective effects through the simultaneous modulation of multiple interconnected pathophysiological pathways, rather than solely by affecting a single molecular target. From a clinical perspective, the results of available studies indicate that appropriately tailored dietary patterns, particularly the Mediterranean diet and the MIND diet, may represent supportive components of preventive strategies and multidisciplinary care; evidence for therapeutic efficacy in established AD remains insufficient. Their potential effectiveness stems from the simultaneous reduction in oxidative stress, modulation of the inflammatory response, and beneficial effects on the composition and metabolic activity of the gut microbiota. However, the current state of knowledge does not yet allow for the formulation of clear clinical recommendations. The results of interventional studies remain inconclusive due to differences in the duration of the intervention, the composition of the diets used, the stage of disease progression, and the significant heterogeneity of the studied populations. It is also still unknown which patient groups may benefit most from specific nutritional interventions. Emerging evidence indicates that treatment response may depend on genetic factors, metabolic status, and the individual composition of the gut microbiota. Various genes are involved in Alzheimer’s disease. The APOE genotype, particularly the presence of the ε4 allele, may be of particular importance, as it is associated with increased oxidative stress, lipid metabolism disorders, and an increased neuroinflammatory response. Primarily, this factor increases susceptibility to the disease and lowers the age of onset. Therefore, the effectiveness of dietary interventions may differ between carriers and non-carriers of this genetic variant. Future clinical trials should therefore incorporate a multidimensional assessment of the effectiveness of dietary interventions. It seems necessary to simultaneously measure biomarkers of oxidative stress, such as MDA, 4-HNE, F_2_-isoprostanes, and 8-oxo-dG, which will enable an objective assessment of changes occurring at the biological level. Equally important is the analysis of the gut microbiota profile and its metabolites, particularly short-chain fatty acids. This will allow for a better understanding of the mechanisms underlying the interaction between diet and central nervous system function. Future studies should also consider categorizing participants by APOE genotype and systematically monitoring adherence to dietary recommendations using validated dietary assessment tools and, where possible, objective biomarkers of dietary intake. It is also essential to use standardized cognitive endpoints, including both standardized tests.

Despite the growing number of studies indicating a link between the composition of the gut microbiota and the development of Alzheimer’s disease, the available evidence still does not allow for a definitive confirmation of this causal relationship. Most data come from observational studies or animal models and indicate the coexistence of dysbiosis, increased oxidative stress, and neuroinflammatory processes. However, they do not determine whether changes in the microbiota are a factor initiating the development of the disease, or rather a consequence of it, or reflect concomitant metabolic disorders and age-related changes.

It is also important to note that the composition of the gut microbiota is determined by many factors, such as diet, age, medications, comorbidities, and lifestyle, which can complicate the interpretation of results and comparisons between individual studies. Consequently, the current state of knowledge points to the potential involvement of the gut–brain axis in the pathogenesis of Alzheimer’s disease rather than a clearly proven etiological mechanism. Therefore, interventions aimed at modulating the microbiota, including dietary modification and the use of probiotics or prebiotics, should currently be considered promising but experimental strategies that will require confirmation in well-designed, randomized clinical trials. Future studies should not only assess changes in microbiota composition but also analyze their association with biomarkers of oxidative stress, neuroinflammatory processes, and cognitive function assessments, as this will allow for a better determination of whether the observed associations are causal or merely correlational.

## 8. Limitations

Despite the growing body of evidence supporting the role of oxidative stress, dietary factors, and the gut microbiota in AD, several important limitations should be considered when interpreting the available literature. First, much of the current evidence is derived from observational studies and preclinical models, while relatively few large, well-designed randomized controlled trials have evaluated the long-term clinical effects of dietary interventions in patients with AD. Consequently, many proposed mechanisms remain biologically plausible but have not yet been conclusively validated in humans.

Another important limitation is the marked heterogeneity among clinical studies. Differences in participant characteristics, disease stage, APOE genotype, dietary assessment methods, intervention duration, supplement dosage, cognitive outcome measures, and follow-up periods make direct comparisons difficult and may partly explain the inconsistent findings reported across studies. Moreover, adherence to dietary interventions is challenging to assess accurately in older adults, and dietary effects are often influenced by other lifestyle factors, including physical activity, educational level, cardiovascular health, medication use, and socioeconomic status.

The interpretation of studies investigating antioxidant supplementation also requires caution. The bioavailability, metabolism, and tissue distribution of many antioxidant compounds vary substantially between individuals, and beneficial effects observed in experimental models have frequently failed to translate into consistent clinical improvements. Furthermore, the biological effects of isolated supplements may differ considerably from those of naturally occurring dietary patterns, where multiple bioactive compounds interact synergistically.

Similar limitations apply to microbiota-related research. Although emerging evidence suggests that alterations in gut microbial composition may contribute to oxidative stress and neuroinflammation, most mechanistic evidence originates from animal studies. Human studies remain limited, and the gut microbiota is influenced by numerous confounding factors, including diet, age, medications (particularly antibiotics), geographical location, and underlying comorbidities. Additionally, substantial variability exists in probiotic strains, doses, treatment duration, and analytical methodologies, limiting comparisons across studies and preventing firm clinical recommendations.

Finally, this review is narrative in nature rather than a systematic review. Although recent high-quality studies were prioritized, no formal assessment of study quality or risk of bias was performed. Consequently, some degree of selection bias cannot be excluded. Nevertheless, this narrative approach allowed the integration of mechanistic, experimental, and clinical evidence to provide a comprehensive overview of the interactions among oxidative stress, dietary interventions, and the gut–brain axis in Alzheimer’s disease.

## 9. Conclusions

AD is a multifactorial neurodegenerative disorder in which oxidative stress represents a key mechanism linking amyloid pathology, tau dysfunction, neuroinflammation, and mitochondrial impairment. Current evidence supports the potential role of healthy dietary patterns as part of comprehensive strategies for maintaining cognitive health and reducing modifiable risk factors associated with Alzheimer’s disease. However, evidence demonstrating clinically meaningful slowing of disease progression remains limited and requires confirmation in adequately powered randomized controlled trials.

## Data Availability

No new data were created or analyzed in this study. Data sharing is not applicable to this article.
